# Microscale Flow Simulation of Resin in RTM Process for Optical Fiber-Embedded Composites

**DOI:** 10.3390/polym17152076

**Published:** 2025-07-29

**Authors:** Tianyou Lu, Bo Ruan, Zhanjun Wu, Lei Yang

**Affiliations:** 1 State Key Laboratory of Structural Analysis, Optimization and CAE Software for Industrial Equipment, School of Mechanics and Aerospace Engineering, Dalian University of Technology, Dalian 116024, China; 22303200@mail.dlut.edu.cn (T.L.); ruanbo@dlut.edu.cn (B.R.); 2 School of Materials Science and Engineering, Dalian University of Technology, Dalian 116024, China; wuzhj@dlut.edu.cn; 3 School of Fiber Engineering and Equipment Technology, Jiangnan University, Wuxi 214122, China

**Keywords:** intelligent composites, optical fiber, resin transfer molding, resin flow, numerical simulation

## Abstract

By embedding optical fiber sensors into fiber preforms and utilizing liquid molding processes such as resin transfer molding (RTM), intelligent composite materials with self-sensing capabilities can be fabricated. In the liquid molding process of these intelligent composites, the quality of the final product is highly dependent on the resin flow and impregnation effects. The embedding of optical fibers can affect the microscopic flow and impregnation behavior of the resin; therefore, it is necessary to investigate the specific impact of optical fiber embedding on the resin flow and impregnation of fiber bundles. Due to the difficulty of directly observing this process at the microscopic scale through experiments, numerical simulation has become a key method for studying this issue. This paper focuses on the resin micro-flow in RTM processes for intelligent composites with embedded optical fibers. Firstly, a steady-state analysis of the resin flow and impregnation process was conducted using COMSOL 6.0 obtaining the velocity and pressure field distribution characteristics under different optical fiber embedding conditions. Secondly, the dynamic process of resin flow and impregnation of fiber bundles at the microscopic scale was simulated using Fluent 2022R2. This study comprehensively analyzes the impact of different optical fiber embedding configurations on resin flow and impregnation characteristics, determining the impregnation time and porosity after impregnation under different optical fiber embedding scenarios. Additionally, this study reveals the mechanisms of pore formation and their distribution patterns. The research findings provide important theoretical guidance for optimizing the RTM molding process parameters for intelligent composite materials.

## 1. Introduction

In the aerospace sector, achieving lightweight structural designs has been a key focus of research. Achieving lightweight structures not only increases the effective payload capacity of aircraft and extends their range or firing distance but also significantly reduces energy consumption, aligning with the principles of green development. Composite materials, characterized by their integrated molding capabilities, offer high specific stiffness and strength, low density, excellent resistance to corrosion and fatigue, good dimensional stability, and strong design flexibility. These advantages enable a reduction in the number of components and connectors [1], making composites increasingly prevalent in advanced manufacturing sectors such as aerospace, marine engineering, and automotive applications [2,3,4,5,6,7].

With the increasing severity of aircraft service environments, the coupling of extreme operational conditions and complex loading scenarios significantly accelerates the degradation of material performance at both macroscopic and microscopic scales. This degradation can significantly reduce the structural load-bearing capacity, potentially resulting in serious safety incidents. Due to the inherent anisotropy and heterogeneity of composite materials, their damage and failure mechanisms are often highly complex and exhibit nonlinear behavior, rendering conventional predictive methods ineffective. Therefore, the implementation of reliable structural health monitoring (SHM) systems for aircraft has become increasingly essential to ensure operational safety and performance. Among various sensing technologies, fiber optic sensors have emerged as one of the most promising solutions for the SHM of smart composite structures due to their advantages such as compact size, light weight, immunity to electromagnetic interference, and high sensitivity [8,9]. By integrating fiber optic sensors into fiber preforms prior to composite fabrication using liquid molding techniques, it is possible to develop intelligent composite structures that fulfill dual functions—lightweight structural performance and real-time internal condition monitoring—thus significantly enhancing the safety, durability, and reliability of high-performance equipment in aerospace and other demanding applications.

The resin transfer molding (RTM) process, recognized for its combination of high-quality output and cost-effectiveness, has been widely adopted across various industrial sectors for the fabrication of composite materials [10]. However, as a closed-mold manufacturing technique, the quality of RTM-fabricated components is highly dependent on the degree of resin infiltration into the fiber preform. During the mold filling stage, non-uniform resin flow or incomplete impregnation can easily lead to defects such as dry spots, pores, and air entrapments [11], all of which compromise the mechanical performance and reliability of the final composite part. Furthermore, when optical fibers are embedded into the fiber preform for sensing or monitoring purposes, the situation becomes more complex. The diameter of optical fibers is typically one order of magnitude larger than that of conventional reinforcing fibers such as carbon or glass fibers. This significant size mismatch can alter the local flow paths of the resin and hinder uniform infiltration. Therefore, investigating the influence of embedded optical fibers on the micro-scale resin flow behavior is of great significance for optimizing the RTM process and improving the quality and functionality of intelligent composite materials.

At present, certain achievements have been made in the research on the mold filling process of resin transfer molding (RTM). Many scholars [12,13,14] have conducted extensive studies on resin flow behavior at the macro-scale. However, these macro-scale investigations are often insufficient to accurately predict the mechanisms and locations of defects such as bubbles and dry spots during the filling process. In terms of micro-scale research, Chen et al. [15,16] introduced the disorder of micro-geometric structures into the model and studied the flow characteristics of a large number of randomly distributed fiber arrays through numerical simulations. It was found that there is a direct correlation between the permeability and the mean nearest inter-fiber spacing, reflecting the influence of the actual material micro-structure on the permeability characteristics. Hiremath et al. [17] developed a model based on Darcy’s law with consideration of resin sorption effects to investigate the influence of injection pressure, permeability, porosity, viscosity, and injection radius on the progression of the resin flow front. Bechtold et al. [18] investigated how the non-uniform distribution of fiber filaments within the fiber bundle affects transverse permeability and clearly pointed out that the real arrangement of filaments deviates from the idealized hexagonal structure. They emphasized the necessity of incorporating realistic filament distributions in geometric modeling. Yang et al. [19] constructed a fiber structure using a Monte Carlo random algorithm and predicted its permeability through numerical simulation. Dong et al. [20] established unit cell models of braided fibers at both the microscopic and mesoscopic scales and simulated the resin flow behavior. Tan et al. [21] proposed a multi-scale model that considers the influence of resin micro-flow behavior on the macro-scale flow. Xiao et al. [22] developed a multi-scale model covering the entire process—from the anisotropic permeability of the meso-scale braided structure to the macro-scale resin infiltration—thus improving the simulation accuracy of the RTM process through multi-scale coupling analysis. In terms of numerical simulation methods for microscale flows, Meghdadi Isfahani [23] has conducted in-depth research on the thermal flow characteristics in micro–nano porous structures.

However, most of the previous numerical simulations of the micro-scale resin mold filling process in RTM have adopted single-phase flow models. These models typically consider only the unidirectional flow of resin, neglecting the presence and influence of air. They often assume that the unfilled regions are directly connected to exhaust vents, with the pressure at the resin flow front defaulted to zero. In reality, during the mold filling process, air may be trapped and wrapped by the advancing resin, forming bubbles whose internal pressure changes dynamically with the flow process. As a result, the pressure at the flow front is not necessarily zero, and single-phase models fail to accurately capture the true behavior of the resin flow. Although Chui et al. [24] proposed a two-phase flow model for the RTM resin filling process, and Park et al. [25] systematically modeled the mechanism and evolution of bubble formation in liquid composite molding, revealing the physical relationship between pore generation and injection parameters through numerical simulation, the current research on two-phase flow still has limitations. In particular, the influence of embedded optical fibers on micro-scale resin flow remains insufficiently understood. Moreover, in the RTM process, pore defects significantly impact the final quality of composite components. It has been shown that mechanical properties such as shear strength, tensile strength, and flexural strength decline with increasing pore content [26]. In light of these issues, this study addresses the gas–liquid two-phase flow characteristics of the RTM mold filling process, with full consideration given to the impact of embedded optical fibers on resin and air interaction at the micro-scale. Through both steady-state and transient simulations, the mechanisms of bubble generation, growth, and the formation of pore defects under the influence of embedded optical fibers are systematically analyzed. The results aim to provide a theoretical basis and technical guidance for improving the molding quality of intelligent composite structures with embedded optical fibers in RTM applications.

## 2. Steady-State Analysis

This section conducts a systematic, steady-state numerical analysis of resin flow to comprehensively investigate the influence of optical fiber embedding on the resin transfer molding (RTM) process. By constructing a micro-structural model with embedded optical fibers, this study aims to characterize the velocity field and pressure distribution of the resin both within and around the fiber bundle, with particular emphasis on the regions adjacent to the optical fibers. This analysis reveals how embedded optical fibers disrupt local flow characteristics—such as velocity perturbation and pressure gradient variation—but also provides critical boundary conditions and initial theoretical insights for the subsequent transient simulation of the mold filling process. Specifically, the obtained steady-state results lay a solid theoretical foundation for in-depth exploration of the resin infiltration mechanism and the underlying pore formation mechanisms, thereby bridging the gap for more accurate prediction of RTM process dynamics.

### 2.1. Model Establishment

**Figure 1:**
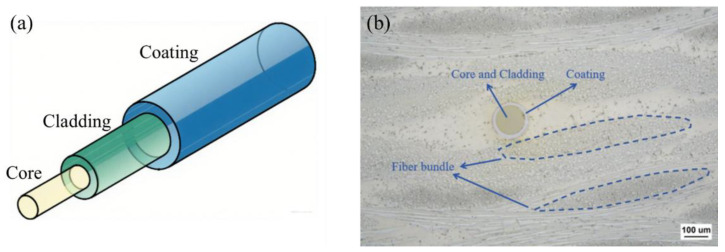
(**a**) Schematic diagram of optical fiber structure and (**b**) microscopic morphology of optical fiber embedded in woven composites.

Figure 1 shows the schematic diagram of the optical fiber structure and the microscopic structure diagram of the composite material with embedded optical fibers. The fiber optic sensor is mainly composed of a core, a cladding, and a coating. After embedding the optical fiber into the woven composite material, the actual micro-structure is very complex due to the deflection and deformation of the fiber bundle caused by extrusion.

**Figure 2:**
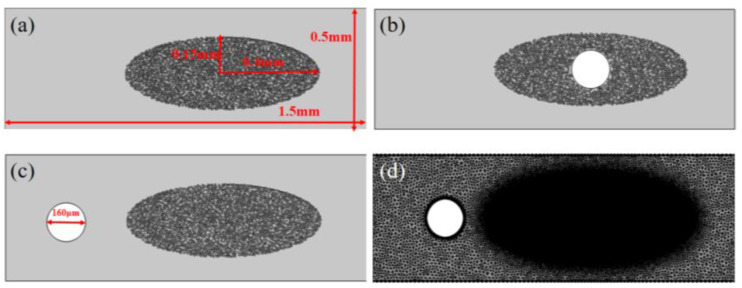
Models of different optical fiber embedding scenarios: (**a**) without optical fiber; (**b**) optical fiber embedded inside the fiber bundle; (**c**) optical fiber embedded outside the fiber bundle; and (**d**) finite element discretization of model.

This paper simplifies the micro-structure of the composite material with embedded optical fibers, only considers the flow of the resin near a fiber bundle, and simplifies the shape of the fiber bundle into an ellipse. Since the resin cannot penetrate into the fiber filaments and the optical fiber, the optical fiber and the fiber filaments are considered as holes. Due to the random distribution of the filaments in the fiber bundle, a fiber bundle structure that conforms to the actual arrangement is generated in COMSOL 6.0 through the Monte Carlo random algorithm, as shown in Figure 2a. The long axis length of the fiber bundle is 0.8 mm; the short axis length is 0.3 mm; the fiber bundle comprises 2700 filaments (7 μm diameter) with a 60% volume fraction. In order to consider the influence of different optical fiber embedding positions on the resin flow, optical fibers with a diameter of 160 μm are, respectively, embedded inside and outside the fiber bundle, as shown in Figure 2b,c. The model was discretized using free triangular elements. Since the flow channel inside the fiber bundle is narrow, in order to ensure the calculation accuracy and convergence, the internal area of the fiber bundle needs to be locally refined. Taking the case without embedded optical fiber as an example, the specific meshing situation is shown in Figure 2d, and the total number of elements is 590,706.

For the resin flow at the micro-scale, based on the characteristic that the entire flow domain conforms to laminar flow, the tracking of the flow front adopts the phase-field method, which obeys the following Cahn–Hilliard equation [27].

(1) $$\frac{\partial \phi }{\partial t}+u\cdot \nabla \phi =\nabla \cdot \frac{\gamma \lambda }{\tau^{2}}\nabla \psi$$

(2) $$\psi =-\nabla \cdot \tau^{2}\nabla \phi +(\phi^{2}-1)\phi$$

In the formula, ϕ is the phase field variable, ψ is the transition variable, λ is the mixed energy density, γ represents the surface tension between the resin and the gas, and the value of τ directly affects the diffusion behavior of the phase field, that is, the transition rate of the phase interface from one phase to another.

Equation (1) is an equation describing the evolution of the phase field variable. The phase field variable represents the phase state distribution of the two-phase fluid, and different phases are usually distinguished by 0 and 1. The core role of this equation is to track the position of the phase interface in the flow. Equation (2) is mainly used to describe the transition variable of the phase field ψ. The transition variable determines the thickness, smoothness, and movement mode of the interface, and it is related to the change of the phase field variable ϕ, reflecting the characteristics of the interface transition.

In addition, during the process of resin flow and infiltration into the fiber bundle, surface tension is an important physical quantity. Especially at the micro-scale, surface tension has a direct impact on the infiltration behavior of the resin and the formation of bubbles.

The expression of surface tension *F*_st_ in the phase field method is

(3) $$F_{\mathrm{st}}=(G-\frac{\partial f}{\partial \phi })\nabla \phi$$

(4) $$G=\lambda (-\nabla^{2}\phi +\frac{\phi (\phi^{2}-1)}{e^{2}})+\frac{\partial f}{\partial \phi }$$

In the formula, *G* is the chemical potential, which represents the interaction energy between the resin and the air.

**Figure 3:**
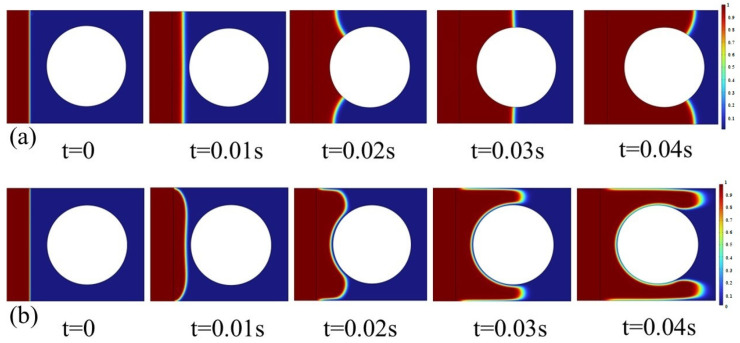
Comparison of resin flow behavior: (**a**) larger surface tension and (**b**) smaller surface tension.

To show the effect of surface tension on resin flow, we analyzed the resin flow behavior in the vicinity of a single fiber filament. Figure 3a depicts the resin flow process past the fiber filament under the condition of high surface tension, whereas Figure 3b shows the corresponding result under low surface tension. As can be observed from the figure, a larger surface tension coefficient makes the resin flow front tend to form a shape with smaller curvature, and the interface exhibits relatively stronger resistance to deformation. In contrast, a smaller surface tension may give rise to intense fluctuations in the flow front. Hence, surface tension plays a crucial role in regulating the interface morphology and stability.

### 2.2. Calculation Results and Analysis

In COMSOL, the steady-state simulation analysis of the flow domain is carried out through the coupling of the Brinkman equation and the fluid phase field. The left boundary of the model is set as the inlet, the inlet pressure is 0.25 MPa, the right side is the outlet, the outlet pressure is 0.1 MPa, and the upper and lower boundaries are non-passable boundaries. The viscosity of the resin is 0.05 Pa·s, and the density is 1300 kg/m^3^.

**Figure 4:**
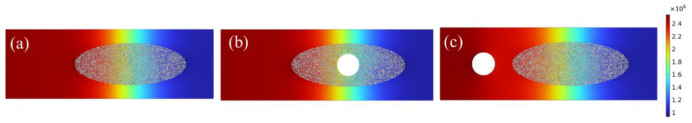
Pressure distribution in the flow domain (unit: Pa): (**a**) without optical fiber; (**b**) optical fiber embedded inside the fiber bundle; and (**c**) optical fiber embedded outside the fiber bundle.

Figure 4 shows the pressure distribution in the flow domain obtained by steady-state calculation under three different optical fiber embedding conditions. It can be seen that when no optical fiber is embedded, the pressure distribution in the flow domain is relatively uniform. When the optical fiber is embedded inside the fiber bundle, the pressure distribution changes significantly: The pressure field around the optical fiber is disturbed, and the area with obvious pressure gradient changes appears, indicating that the optical fiber has obstructed the resin flow. When the optical fiber is embedded outside the fiber bundle, a local high-pressure phenomenon appears at the inlet due to the obstruction of the optical fiber, and a relatively low-pressure area is formed between the optical fiber and the fiber bundle.

**Figure 5:**
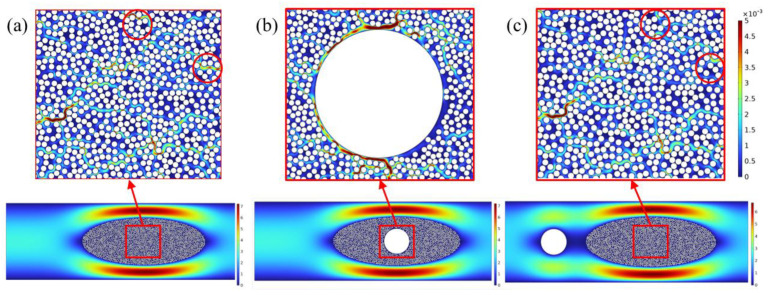
Resin flow velocity distribution (unit: m/s): (**a**) without optical fiber; (**b**) optical fiber embedded inside the fiber bundle; and (**c**) optical fiber embedded outside the fiber bundle.

Figure 5 presents the resin velocity distributions within the flow domain under three different optical fiber embedding configurations. As shown in Figure 5, when no optical fiber is embedded, the resin flows more rapidly along the outer sides of the fiber bundle, with a symmetric velocity distribution observed above and below the bundle. In contrast, due to the narrower flow channels inside the fiber bundle, the resin experiences greater resistance, resulting in significantly lower flow velocities within the bundle. When the optical fiber is embedded inside the fiber bundle, the velocity distribution outside the bundle remains similar to that without fiber embedding; however, noticeable changes in velocity occur in the vicinity of the embedded fiber within the bundle. In the case where the optical fiber is embedded on the outer side of the fiber bundle, the internal flow velocity resembles that of the case without fiber embedding. Nonetheless, the presence of the fiber obstructs the flow, forming a distinct low-velocity region downstream of the fiber. This may lead to inadequate resin infiltration within the fiber bundle and increase the risk of void formation. There are slight differences between Figure 5a–c. The primary reason is that while the flow domain has reached a steady state, the resin flow velocity within the fiber bundle is inherently very low, which leads to only minor discrepancies between the two figures. Nevertheless, some differences do exist, as highlighted by the red circles.

To verify the correctness of our method, we calculated the permeability of a fiber bundle model and compared the results with those reported in the existing literature. The fiber bundle has a major axis length of 4.4 mm and a minor axis length of 0.5 mm, containing 489 individual fibers. As illustrated in Table 1, the permeability result obtained in this study is 2.18823×10^−8^, with relative errors of 4.59% and 6.43% compared to two separate references, respectively. These validation results demonstrate the rationality and reliability of the method employed in this research.

## 3. Transient-State Analysis

The steady-state calculation can obtain the pressure and velocity distribution of the resin in the flow domain, but the specific flow process of the resin in the flow domain is still unknown, especially when the optical fiber is embedded; the flow situation of the resin will be affected by the optical fiber. Therefore, in order to study the flow behavior of the resin during the infiltration process and the generation and elimination process of bubbles, this section carries out a transient simulation of the resin flow process in Fluent 2022R2.

### 3.1. Transient Model

For the phase interface between the gas and liquid phases during the flow process, since it moves and changes shape with the continuous flow of the resin, how to accurately track the interface between the two phases is the most critical. At present, the common multi-phase flow interface tracking methods mainly include the Front Tracking method, the Level Set method, the phase field method, and the VOF method (Volume of Fluid), etc. Among these, the Volume of Fluid (VOF) method is a widely adopted phase interface tracking method, proposed by Hirt and Nichols [30]. This method tracks the fluid interface by monitoring the volume fraction within each computational cell, offering high accuracy, ease of implementation, and computational efficiency [31]. Therefore, this paper uses the VOF method to track the two-phase flow interface.

The core of the VOF method lies in defining the phase function *F* to represent the volume fraction of the fluid in the flow field. The flow situation of the resin in the entire flow field can be roughly divided into three types. When *F* = 0, it indicates that there is no resin in the area, and the inside is all air phase; when 0 < *F* < 1, there are both resin and air in the area, and the phase interface between the gas and liquid phases is in this area; when *F* = 1, the inside of the area is filled with resin, and there is no air.

The change of the volume fraction *F* with time and space is described by the transport equation:

(5) $$\frac{\partial F}{\partial t}+u\cdot \nabla F=0$$

In the equation, *t* is time, and ***u*** is the velocity vector.

This equation is essentially the embodiment of the mass conservation equation in the VOF method, and its physical meaning is as follows: The change of the fluid volume fraction in the calculation unit per unit time is equal to the net flux of the fluid volume fraction flowing in and out through the boundary of the unit.

Assuming that the resin is an incompressible fluid, the continuity equation in the multi-phase flow process can be expressed as:

(6) ∇u=0

In Equation (5), the physical properties of the fluid are determined by the ratio of the volume of each phase fluid in each control volume to the volume of the control volume. In order to obtain the viscosity of the fluid in the gas–liquid two-phase flow model, it is assumed that the volume fraction of the liquid phase is known, then the viscosity formula and density formula in the control volume are as follows:

(7) $$\mu =F\mu_{l}+(1-F)\mu_{g}$$

(8) $$\rho =F\rho_{l}+(1-F)\rho_{g}$$

In the formula, µL and *ρ_l_* represent the viscosity and density of the resin, respectively, while *µ_g_* and *ρ_g_* represent the viscosity and density of the gas, respectively.

The VOF method uses the volume integral form to describe the momentum change of the fluid in the control unit, and the velocity magnitude is obtained through the momentum conservation equation in the unit. The momentum control equation of the gas-liquid two-phase is:

(9) $$\frac{\partial (\rho_{f}u)}{\partial t}+\nabla \cdot (\rho_{f}u\times u)=-\nabla p+\mu_{f}\nabla u+\rho_{f}f$$

In the formula, the parameters with the subscript *f* represent the physical parameters of the fluid, which can be the resin, the air, or the mixed part of the resin and the air. This equation is the core part of the VOF method and plays a key role in simulating the interaction of each interface in the multi-phase flow and the velocity jump at the interface.

In this paper, the SIMPLEC algorithm is used for the coupling problem of pressure and velocity. This algorithm is based on the pressure correction method. It first makes an initial prediction of the velocity field and pressure field and then corrects them through step-by-step iteration to make the velocity field and pressure field satisfy the continuity equation and momentum equation. For complex flow problems, especially multi-phase flow problems, it can achieve the convergence requirement with fewer iterations.

**Figure 6:**
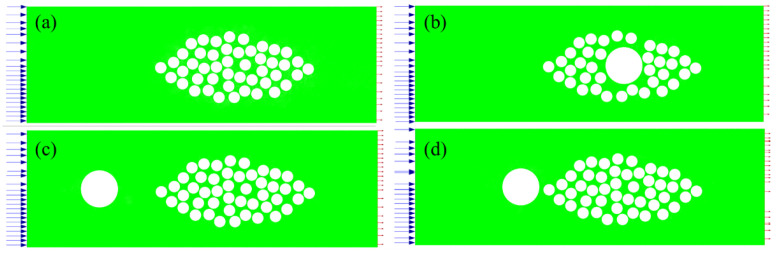
Transient model: (**a**) without optical fiber; (**b**) optical fiber embedded inside the fiber bundle; (**c**) optical fiber embedded outside and far from the fiber bundle; and (**d**) optical fiber embedded outside and near the fiber bundle.

For transient calculations, as the grid size decreases, in order to meet the numerical stability, the time step must be correspondingly reduced, which will significantly increase the total calculation time. In order to improve the calculation convergence degree and reduce the calculation time, the fiber bundle model is simplified. On the premise of ensuring that the fiber volume fraction remains unchanged, the radius of the fiber filaments is increased to 25 µm, and other parameters are kept consistent with the steady-state model. The model is shown in Figure 6. In addition, in order to further illustrate the influence of the optical fiber embedding position on the resin flow process, a model in which the optical fiber is embedded in a position close to the outside of the fiber bundle is added, as shown in Figure 6d.

### 3.2. Grid Independence Verification

In numerical simulations, both the quantity and quality of the mesh elements within the flow domain play a crucial role in determining the accuracy of the simulation results. In general, a higher grid quality combined with a greater number of grid elements tends to produce results that more closely approximate real physical behavior. However, increasing the number of grids also leads to a significant rise in computational complexity and simulation time, which can become a limiting factor, especially in large-scale simulations. Therefore, it is essential to develop a grid model that minimizes the number of elements while still maintaining sufficient accuracy in order to achieve an effective balance between computational efficiency and result reliability.

**Figure 7:**
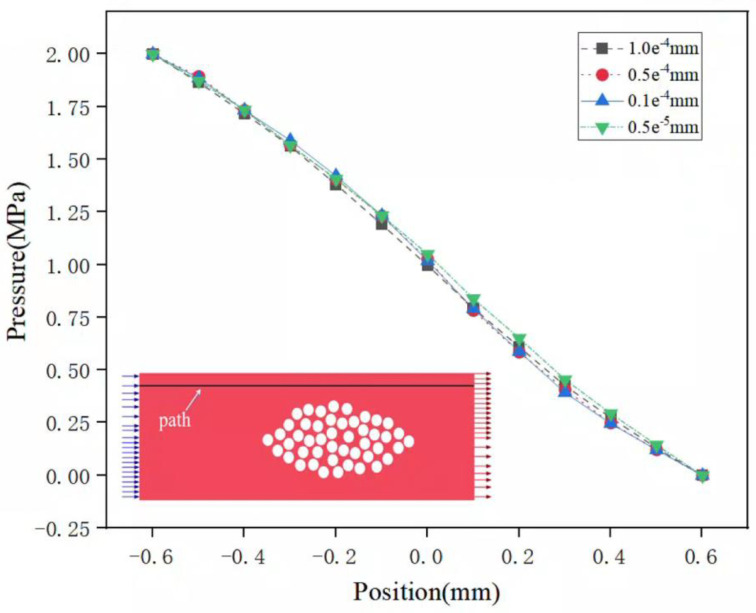
Pressure distribution along the monitoring path.

In order to determine the most appropriate grid size, the flow field is simulated under different mesh resolutions, and the grid independence is verified. Four groups of grids with grid sizes of 1 × 10^−4^ mm, 0.5 × 10^−4^ mm, 0.1 × 10^−4^ mm, 0.5 × 10^−5^ mm are, respectively, divided, and the four groups of grids are sequentially subjected to steady-state analysis. Under the same boundary conditions, the pressure on a path inside the flow domain (as shown in Figure 7) is monitored, and the influence of different grid sizes on the simulation results is compared.

**Figure 8:**
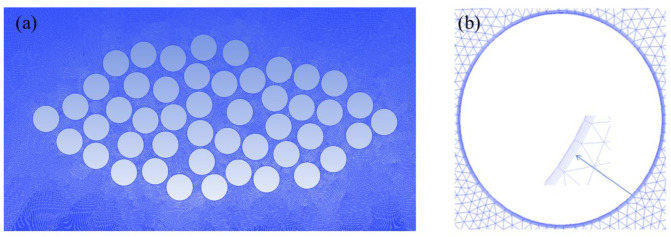
Schematic of mesh discretization. (**a**) Mesh distribution of the entire domain. (**b**) Schematic of boundary layer meshing.

The calculated pressure distribution is shown in Figure 7. It can be seen that the pressure distribution of the monitoring path under different grid sizes is basically the same, indicating that different grid sizes have little influence on the calculation results. Therefore, a triangular mesh with a size of 1 × 10^−4^ mm is selected for discretizing the model. In addition, to ensure calculation accuracy and convergence, boundary layer meshing was performed on both the fiber filaments and the vicinity of the optical fibers. The number of boundary layers is 6, the thickness of the innermost grid is 1 × 10^−5^ mm, and the growth rate is set to 1.1. Figure 8a shows the meshing situation of the model without embedded optical fibers, the total number of grids is 798,922, and Figure 8b shows the boundary layer grid situation.

### 3.3. Results and Discussion

**Figure 9:**
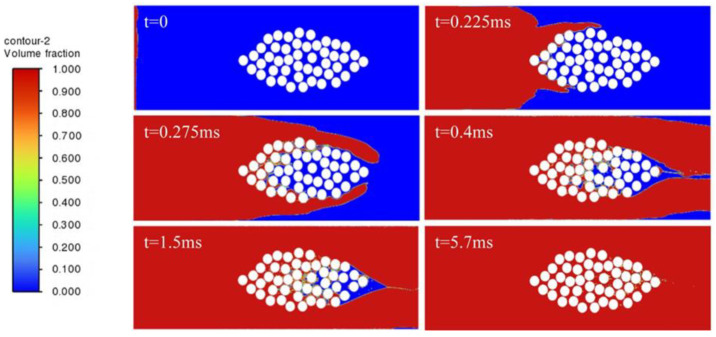
Resin infiltration process without embedded optical fiber.

In order to study the influence of embedded optical fibers on the resin infiltration process of the fiber bundle, the cases of no optical fiber and optical fibers embedded in different positions are compared. The resin infiltration process without embedded optical fibers is shown in Figure 9. The resin flow velocity outside the fiber bundle is fast, while inside the fiber bundle, due to the obstruction of the fiber filaments and the narrow flow channel, the resin flow velocity is relatively slow. The resin outside converges near the outlet, encapsulating the internal gas. As the resin is continuously injected, the inside of the fiber bundle is continuously filled, most of the area of the fiber bundle is infiltrated, but tiny bubbles will be formed. Most of the bubbles can be eliminated after a long time of mold filling.

**Figure 10:**
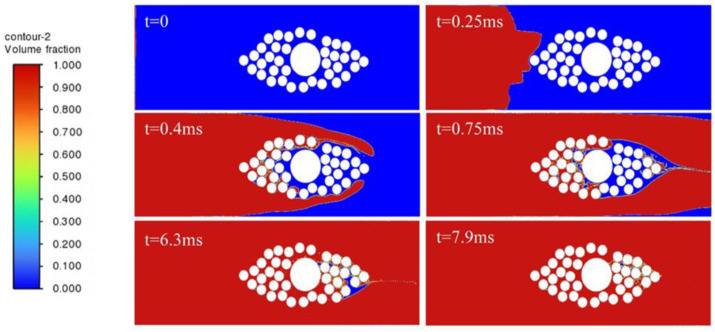
Resin infiltration process with optical fiber embedded inside the fiber bundle.

Figure 10 shows the process of resin infiltration into the fiber bundle when the optical fiber is embedded inside the fiber bundle. It can be observed that compared with the infiltration situation of the fiber bundle without embedded optical fibers, due to the obstruction of the optical fiber at the center of the fiber bundle, when the resin outside flows to the rightmost side of the fiber bundle to form a wrapping, the un-infiltrated area inside the fiber bundle is larger. Due to the presence of the optical fiber, bubbles are more likely to form on the right side of the optical fiber, and it takes significantly longer for the resin to infiltrate the area downstream of the optical fiber.

**Figure 11:**
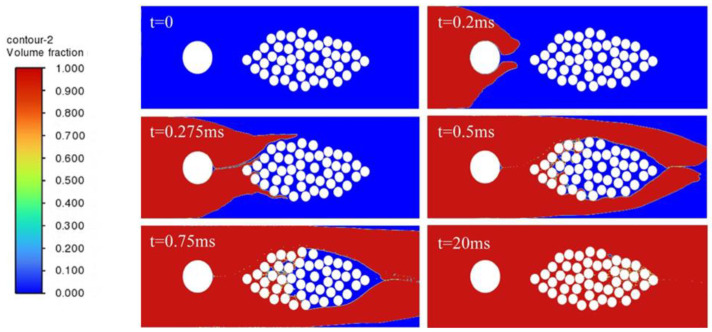
Resin infiltration process with optical fiber embedded at a distant outer side of the fiber bundle.

Figure 11 shows the process of resin flow and infiltration into the fiber bundle when the optical fiber is embedded in a position far outside the fiber bundle. Compared with the case without embedded optical fibers, due to the presence of the optical fiber, the embedded optical fiber will make the resin form a larger wrapping around the fiber bundle. When the resin outside forms a wrapping around the fiber bundle, only a small amount of resin flows into the inside of the fiber bundle, and the resin is hindered from entering the inside of the fiber bundle, and a required infiltration time is greatly prolonged.

**Figure 12:**
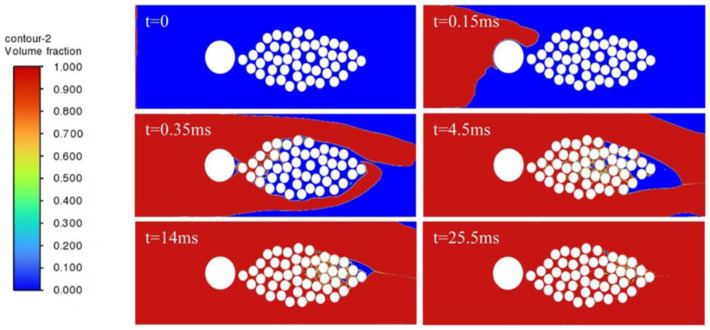
Resin infiltration process with optical fiber embedded at a nearby outer side of the fiber bundle.

Figure 12 shows the process of resin infiltration into the fiber bundle when the optical fiber is embedded in a position close to the outside of the fiber bundle. The results show that when the optical fiber is close to the fiber bundle, bubbles and insufficient infiltration are likely to occur in the middle position between the optical fiber and the fiber bundle. The resin infiltration into the fiber bundle is significantly hindered, and the resin outside almost forms a wrapping around the entire fiber bundle. The infiltration of the fiber bundle takes significantly more time, and after a long time of mold filling, a small number of bubbles still exist inside the fiber bundle, which indicates that the embedded optical fiber has a great influence on the infiltration of the fiber bundle.

**Figure 13:**
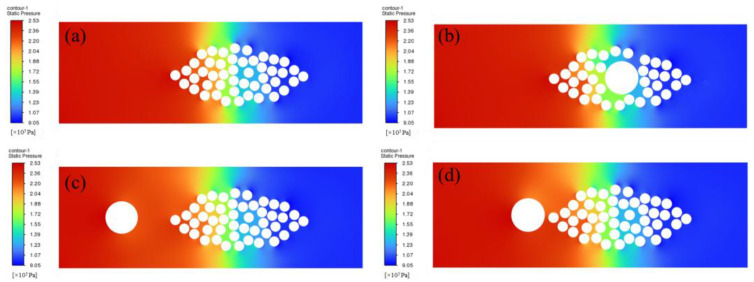
Pressure distribution in the flow domain during transient analysis: (**a**) without optical fiber; (**b**) optical fiber embedded inside the fiber bundle; (**c**) optical fiber embedded outside and far from the fiber bundle; and (**d**) optical fiber embedded outside and near the fiber bundle.

Figure 13 shows the pressure distribution in the flow domain after the resin is completely filled. When no optical fiber is embedded, the pressure distribution is relatively uniform, and the transition from the high-pressure area to the low-pressure area is smooth; when the optical fiber is embedded inside the fiber bundle, due to the presence of the optical fiber, the pressure distribution near the optical fiber is disturbed, and the degree of change is large; when the optical fiber is embedded outside the fiber bundle, due to the obstruction of the optical fiber, a low-pressure transition area will be formed on the right side of the optical fiber. Compared with the pressure results obtained from the above steady-state calculation, the overall trend is relatively close, thereby verifying the correctness of the simulation results.

There are two main types of pressure distribution during the formation of bubbles in the resin filling process:

The first type occurs in the early stage of bubble formation, where the pressure inside the bubble is lower than the external resin pressure. Under the influence of this pressure difference, resin flows into the bubble, causing the bubble size to decrease and the internal pressure to increase, until the internal pressure of the bubble matches the external resin pressure. At this point, the resin no longer flows into the bubble, and the bubble size stabilizes.

The second type involves bubbles being surrounded by resin with varying pressures. The resin gradually flows into the bubble, increasing the internal pressure of the bubble. This continues until the internal pressure of the bubble matches the lowest external resin pressure. Afterward, the resin at the lowest pressure region stops flowing into the bubble, while the resin at the higher-pressure region continues to flow into the bubble. This leads to further increases in the bubble’s internal pressure, which eventually exceeds the pressure in the lowest region of the resin, causing the bubble to overflow from the low-pressure zone.

**Figure 14:**
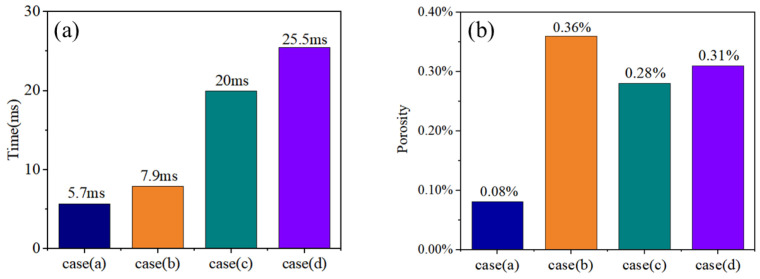
Comparative analysis of resin filling and infiltration results: (**a**) infiltration time; (**b**) porosity.

Figure 14 shows the comparison of the time required for resin to infiltrate the fiber bundle and the porosity after mold filling under different optical fiber embedding methods. Among them, case (a), case (b), case (c), and case (d) are the cases of no embedded optical fiber, optical fiber embedded inside, optical fiber embedded far outside the fiber bundle, and optical fiber embedded close outside the fiber bundle, respectively. The results show that in the case of no embedded optical fiber, the time required for the resin to infiltrate the fiber bundle is the shortest, only 5.7 ms. When the optical fiber is embedded inside the fiber bundle, the resin infiltration time is slightly increased, but the difference is relatively small, which is 7.9 ms. However, when the optical fiber is embedded outside the fiber bundle, the required infiltration time is greatly increased to more than 20 ms; especially when the optical fiber is embedded in a position close to the outside of the fiber bundle, the obstruction effect of the optical fiber on the resin is more significant, and the infiltration time reaches 25.5 ms, which is about five times the time required when no optical fiber is embedded.

In terms of porosity, the porosity of the model without embedded optical fiber is the lowest, only 0.08%; the porosity increases after the optical fiber is embedded, especially when the optical fiber is embedded inside the fiber bundle, and the porosity increases most significantly, reaching 0.36%. The main reason for the increase in porosity after the optical fiber is embedded is the uneven pressure distribution in the flow field. When the bubble is wrapped by resins with different pressures, the resin in the high-pressure area continues to flow into the inside of the bubble, gradually increasing the internal pressure until the pressure inside the bubble reaches equilibrium with the lowest external pressure. After that, the resin in the low-pressure area no longer flows in, while the resin in the high-pressure area still continues to flow in, causing the internal pressure of the bubble to exceed the surrounding lowest pressure area, prompting the gas to overflow from the low-pressure area. It can be seen from the above simulation results that when the optical fiber is embedded inside the fiber bundle, the pores are mainly concentrated on the right side of the fiber bundle. The main reason is that the flow channel of the resin inside the fiber bundle is too narrow, and due to the great obstruction of the optical fiber inside the fiber bundle, the flow of the resin is difficult, resulting in the highest porosity.

## 4. Conclusions

This study presents a microscopic-scale simulation of the resin flow and impregnation processes in resin transfer molding (RTM) for composite materials embedded with optical fibers, with a specific focus on analyzing how different fiber embedding configurations impact resin flow and impregnation. First, steady-state simulations were conducted using COMSOL 6.0. The results revealed that when the optical fiber is embedded inside the fiber bundle, significant pressure gradients and uneven distributions form in the vicinity of the fibers. When optical fiber is embedded outside the fiber bundle, the obstruction caused by the fibers creates a distinct local high-pressure zone, and the high-velocity region outside the bundle becomes larger—this may lead to insufficient impregnation within the fiber bundle. These observations necessitated a transient analysis of the impregnation process. Transient simulation results from Fluent 2022R2 demonstrated that embedding optical fibers significantly affects both the impregnation time and the final porosity of the molded part. Without embedded optical fiber, the infiltration time is only 5.7 ms, with the lowest porosity of 0.08%. With embedded optical fiber, both infiltration time and porosity increase to varying degrees. Under identical pressure conditions, embedding the optical fiber closer to the outside of the fiber bundle results in the longest infiltration time (25.5 ms), approximately five times longer than the scenario without embedded fiber. When optical fiber is embedded inside the fiber bundle, the maximum porosity reaches 0.36%. These findings indicate that different optical fiber embedding configurations directly influence resin impregnation quality and, consequently, the final molding quality of composite materials.

Based on these results, this study provides important theoretical guidance for optimizing the RTM process. By designing appropriate optical fiber embedding schemes, the porosity of molded parts can be reduced, thereby enhancing the overall performance of composite materials. Furthermore, this research offers technical support for manufacturing composite materials in high-demand fields such as aerospace, providing theoretical references for optimizing composite material molding processes.

## Figures and Tables

**Table 1:** Comparison of permeability (unit: m^2^).

	Current	Bibliography [28]	Bibliography [29]
**Permeability**	2.18823 × 10^−8^	2.2886 × 10^−8^	2.3289 × 10^−8^

## Data Availability

The raw data supporting the conclusions of this article will be made available by the authors on request.
